# New Approaches to Critical Illness Polyneuromyopathy: High-Resolution Neuromuscular Ultrasound Characteristics and Cytokine Profiling

**DOI:** 10.1007/s12028-020-01148-2

**Published:** 2020-11-24

**Authors:** Anna Lena Fisse, Caroline May, Jeremias Motte, Xiomara Pedreiturria, Thomas G. K. Breuer, Christiane Schneider-Gold, Katrin Marcus, Ralf Gold, Min-Suk Yoon, Kalliopi Pitarokoili

**Affiliations:** 1 Department of Neurology, St. Josef-Hospital, Ruhr-University Bochum, Gudrunstrasse 56, 44791 Bochum, Germany; 2 Medizinisches Proteom-Center, Ruhr-University Bochum, Bochum, Germany; 3 Department of Internal Medicine I, St. Josef-Hospital, Ruhr-University Bochum, Bochum, Germany; 4 Department of Neurology, Evangelisches Krankenhaus Hattingen, Hattingen, Germany

**Keywords:** Intensive care unit acquired weakness, Critical illness polyneuropathy, Critical illness myopathy, Critical illness polyneuromyopathy, Nerve ultrasound, Cytokines

## Abstract

**Background:**

Diagnosis of intensive care unit acquired weakness (ICUAW) is challenging. Pathogenesis of underlying critical illness polyneuromyopathy (CIPNM) remains incompletely understood. This exploratory study investigated whether longitudinal neuromuscular ultrasound examinations and cytokine analyses in correlation to classical clinical and electrophysiological assessment contribute to the understanding of CIPNM.

**Methods:**

Intensive care unit patients were examined every 7 days until discharge from hospital. Clinical status, nerve conduction studies, electromyography as well as ultrasound of peripheral nerves and tibial anterior muscle were performed. Cytokine levels were analyzed by a bead-based multiplex assay system.

**Results:**

Of 248 screened patients, 35 patients were included at median of 6 days (IQR: 8) after admission to intensive care unit. Axonal damage was the main feature of CIPNM. At the peak of CIPNM (7 days after inclusion), nerve ultrasound showed cross-sectional area increase of tibial nerve as a sign of inflammatory edema as well as hypoechoic nerves as a possible sign of inflammation. Cytokine analyses showed signs of monocyte and macrophage activation at this stage. Fourteen days after inclusion, cytokines indicated systemic immune response as well as profiles associated to neovascularization and regeneration.

**Conclusions:**

Exploratory neuromuscular ultrasound and cytokine analyses showed signs of inflammation like macrophage and monocyte activation at the peak of CIPNM followed by a systemic immune response parallel to axonal damage. This underlines the role of both axonal damage and inflammation in pathogenesis of CIPNM.

**Electronic supplementary material:**

The online version of this article (10.1007/s12028-020-01148-2) contains supplementary material, which is available to authorized users.

## Introduction

Intensive care unit acquired weakness (ICUAW) is a frequent problem in intensive care medicine and occurs in up to 67% of patients who are mechanically ventilated for more than 10 days [1]. The defining clinical symptom of ICUAW is a generalized symmetric muscle weakness, leading to a flaccid tetraparesis [2]. This results in prolonged ventilator dependency, prolonged hospitalization and rehabilitation [3] as well as to worse clinical outcome [4]. Causes of ICUAW are critical illness polyneuropathy and critical illness myopathy or combination of both (CIPNM).

ICUAW is a clinical diagnosis based on assessment of muscle strength. However, in analgosedated patients with impairment of consciousness, sensorimotor symptoms are often not reliably determinable [5]. Therefore, electrophysiological measurements are used for diagnosis. Reliability of these measurements on intensive care units is limited [6]: Amplitudes may be difficult to interpret due to peripheral edema and electromagnetic artifacts of the intensive care unit devices. Also, electrophysiological diagnosis based on a single measurement is challenging as other polyneuropathies, i.e., preexisting or acute axonal polyradiculoneuropathy (Guillain-Barré syndrome) variants are difficult to differentiate from axonal polyneuropathy in CIPNM. These difficulties often lead to delayed diagnosis of ICUAW and CIPNM [2] and stress the need for novel approaches in diagnosing CIPNM. Some studies used high-resolution neuromuscular ultrasound for diagnosis of CIPNM [7] with focus on muscular ultrasound, showing muscular atrophy and increased muscle echogenicity as the main findings [7–9]. Only in one study, morphological changes of peripheral nerves in critical illness polyneuropathy were examined using ultrasound. This study did not show a diagnostic benefit of a single ultrasound examination of the median and fibular nerve in diagnosis of critical illness polyneuropathy [10].

The pathogenesis of critical illness polyneuropathy and polymyopathy remains incompletely understood. Metabolic, ischemic and inflammatory factors as well as oxidative stress are considered as major factors leading to axonal damage and demyelination [1, 11]. Microvascular changes increase vascular permeability and enable penetration of neurotoxic inflammatory factors through the blood–nerve barrier in critical illness polyneuropathy [1]. Proinflammatory mediators like tumor necrosis factor alpha, interleukin-1, procalcitonin, e-selectin, syndecan-1 and interleukin-6, interleukin-8, interleukin-10 and fractalkine/CX3CL1 were described to be involved in pathogenesis in the first days of the disease [12, 13]. On the contrary, other studies showed elevated cytokines and complement activation in both patients with ICUAW and without ICUAW as controls, but not in healthy patients, suggesting that cytokine elevation might be present in intensive care unit patients irrespective of the development of ICUAW [12, 14, 15].

This exploratory study investigated whether longitudinal neuromuscular ultrasound examinations and cytokine analyses in correlation to classical clinical and electrophysiological assessment contribute to the understanding of CIPNM. We examine longitudinal cytokine expression in order to discover specific patterns and enhance the understanding of inflammatory mechanisms in CIPNM. Secondly, we analyzed whether high-resolution neuromuscular ultrasound is helpful in diagnosis and monitoring of CIPNM.

## Methods

### Patients

Newly admitted patients of a neurologic-internal medicine intensive care unit in a university hospital of the Ruhr-University Bochum were screened consecutively between March 2017 and February 2018 for inclusion and exclusion criteria to find patients in risk of developing a CIPNM without preexisting polyneuropathy. Inclusion and exclusion criteria are shown in Table 1. Patients meeting inclusion and exclusion criteria were prospectively examined clinically, electrophysiologically and with neuromuscular ultrasound at inclusion (as early as possible after admission to intensive care unit, baseline) and every 7 days until discharge from the hospital or death. Clinical neurological examination was performed by a medical doctor for neurology with 4 years of experience in neurology (AF) and included testing of vigilance graded with Glasgow Coma Scale, muscle strength assessment graded with Medical Research Council (MRC) sum score, assessment of deep tendon reflexes and, if vigilance was sufficient: cranial nerve examination, sensory system examination (touch, pain, temperature and vibration), cerebellar testing (finger to nose test, ankle over tibia test, diadochokinesis) and speech functions. Serum samples were collected at the same time points for cytokine analysis. ICUAW was defined as proposed in Jonghe et al. [5] using assessment of muscle strength: ICUAW was diagnosed if MRC sum score was < 48 points [5].

**Table 1:** Inclusion and exclusion criteria

Inclusion criteria:
Intensive care unit treatment with
Mechanical ventilation for at least 24 h and/or
Sepsis, defined by sepsis-3 criteria [34] and/or
Acute respiratory distress syndrome, defined by the Berlin definition [35] and/or
Renal replacement therapy and/or
Circulatory failure with catecholamine therapy for at least 24 h
Exclusion criteria:
Preexisting diagnosis of polyneuropathy
HbA1c > 8.5% or preexisting diabetes mellitus
HIV infection
Hepatitis infection
Evidence of excessive alcohol consumption in anamnesis or diagnoses
Any drug addiction
Previous chemotherapy with possible side effect of polyneuropathy (i.e., taxanes, platinum derivatives)
Vitamin B12 deficiency
Severe thyroid disorder (i.e., thyrotoxic crisis or myxedema coma)

### ICU Characteristics

This study was performed at an ICU with internistic and neurologic patients. The ward consists of intermediate care and intensive care unit areas. Standard type of sedation is propofol-sufentanyl, alternatively midazolam-sufentanyl, clonidin/dexmedetomidine-sufentanyl or isofluran-sufentanyl, controlled by Richmond Agitation Sedation Scale, with daily attempts to wake up. Patients are temporarily positioned by the nursing staff in an elevated upper body position, side position, incomplete or complete prone position or by means of continuous lateral rotation therapy (e.g., rotor rest). Physiotherapy is available for all patients daily. Passive Range of Motion (PROM) mobilization is performed during physiotherapy in analgosedated patients and Active Range of Motion (AROM) in patients with sufficient consciousness. Mobilization in a chair according to Bobath is performed in tracheostomized patients. Neuromuscular electrical stimulation or functional electrical stimulation is not used regularly.

### Electrodiagnostics

Patients underwent longitudinal motor nerve conduction studies of one tibial, one fibular, one median and one ulnar nerve as well as sensory nerve conduction studies of one sural nerve. If there were no contraindications (i.e., anticoagulation), a needle electromyography of one anterior tibial muscle was performed. Electrodiagnostics were performed by a board-certified neurologist (KP) using a Natus Dantec Keypoint Focus EMG Device (Natus GmbH, Trier, Germany), version 2.33, on basis of international standards [16]. As reference values, we used the ones proposed from Stöhr et al. [17]. The following parameters were measured and evaluated: distal motor latency, motor conduction velocity, F wave latency, F wave persistency (defined as number of detectable F waves per number of stimulations) and amplitude of compound motor action potential and sensory nerve action potential. Diagnosis of CIPNM was performed based on criteria reported in Stevens et al. [18]. Due to artifacts on intensive care unit and technical issues like edema, sensory nerve conduction studies are not reliable; therefore, we did not perform sensory neurography. Needle electromyography was only performed, if no contraindications like anticoagulation occurred. Muscle or nerve biopsy could not be performed as part of this study. Therefore, we adapted the criteria to the following:

CIPNM was defined as:

- Deterioration of the compound motor action potential amplitude during intensive care unit stay by more than 50% and more than 1 mV compared to the baseline nerve conduction study in at least one leg nerve, or
- Presence of pathological spontaneous activity in the needle electromyography.

This definition was also used in patients without awakening during intensive care unit treatment where muscular assessment is not reliable, i.e., due to sedation and impairment of consciousness.

Due to missing standardized methods for longitudinal analysis of CIPNM severity in nerve conduction study and needle electromyography, the following score was developed (CIPNM severity score):

- Regarding nerve conduction study of the fibular nerve:
- 1 point for demyelinating characteristics (reduced conduction velocity, prolonged distal motor latency, prolonged F wave latency, conduction block)
- 1 point for distal compound motor action potential amplitude reduction below the lower limit of normal
- 1 additional point for distal compound motor action potential amplitude reduction > 20%
- 1 additional point for distal compound motor action potential amplitude reduction > 50%
- 1 additional point for distal compound motor action potential amplitude reduction > 70%
- 1 additional point for lack of distal compound motor action potential
- Regarding needle electromyography of the tibialis anterior muscle:
- 1 point for pathological spontaneous activity in ≤ 5 needle layers
- 2 points for pathological spontaneous activity in > 5 needle layers.

CIPNM in our cohort was best identified by nerve conduction studies of fibular nerve; hence, we used nerve conduction studies of fibular nerve for CIPNM severity score.

### High-Resolution Ultrasound

Ultrasound was performed at the same day with the electrophysiology from a neurologist with neuromuscular ultrasound experience (AF). All ultrasound studies were performed with the use of an Affinity^®^ 70G ultrasound system (Philips, Hamburg, Germany). Ultrasound was performed according to the previously described protocol [19]. Additionally, vagal nerve in carotid sheath was investigated. An 18 MHz linear array transducer was used for nerve and muscle ultrasound. Ultrasound settings (e.g., contrast) excluding depth and focus were kept constant during all examinations. Dynamic range was set at 55 dB. The transducer was always kept perpendicular to the nerves, and no additional force was applied other than the weight of the transducer. Cross-sectional area measurements were performed at the inner border of the hyperechoic epineural rim by manual continuous tracing technique. As reference values for cross-sectional area, the ones published by Kerasnoudis et al. [20] were used. Echogenicity was calculated as previously described [21] via semi-automated and quantitative analysis of fraction of black using ImageJ (National Institutes of Health, Bethesda, Maryland, USA), version 1.5.1.

Muscle ultrasound was performed of one tibial anterior muscle at the first third between knee and malleolus lateralis in an axial section. Muscle echogenicity was graded with a score from 1 (normal echogenicity with distinct bone echo) to grade 4 (increased echo intensity and loss of bone signal) according to Heckmatt et al. [22].

### Cytokine Analysis

Cytokine analyses were performed blinded to the clinical data. Serum samples were analyzed at inclusion, seven and 14 days after inclusion. Blood was collected in a serum vacutainer. Serum was obtained from whole blood by centrifuging at 2000 g for 15 min at 4 °C. Then aliquots were frozen and stored at − 80 °C within 15 min. Cytokines were analyzed by a bead based multiplex assay system (Platinum ProcartaPlex™ Immunoassay, Thermo Fisher Scientific, Waltham, Massachusetts, USA) according to the manufacturer’s protocol. Analyzed cytokines included in the multiplex panel and their abbreviations are listed in supplementary Table 1. As we assumed that there are inflammatory processes that decrease or are counter-regulated in the course of the disease, we have selected a standard multiplex panel that contains both pro- and anti-inflammatory cytokines. The exact composition of the panel was given by the manufacturer.

### Statistics

Statistics were performed using GraphPad Prism 8 (GraphPad Software Inc., San Diego, California, USA) and IBM SPSS Statistics 25.0.0.0 (IBM Corporation, Armonk, New York, USA). Absolute data are presented as mean ± SD or as median with IQR. Differences between groups were tested by Mann–Whitney*U* test, *t* test or Chi squared-test as applicable. Benjamini–Hochberg procedure was used to decrease the false discovery rate of multiple comparisons for non-exploratory clinical data. For exploratory data on ultrasound, nerve conduction parameters and cytokines, an appropriate multiple test adjustment was not performed according to Bender et al. [23]. Probability levels (*p*-values) are indicated as *, if *p* ≤ 0.05, as **, if *p* ≤ 0.01, and as ***, if *p* ≤ 0.001.

## Results

### Clinical Data

**Fig. 1:**
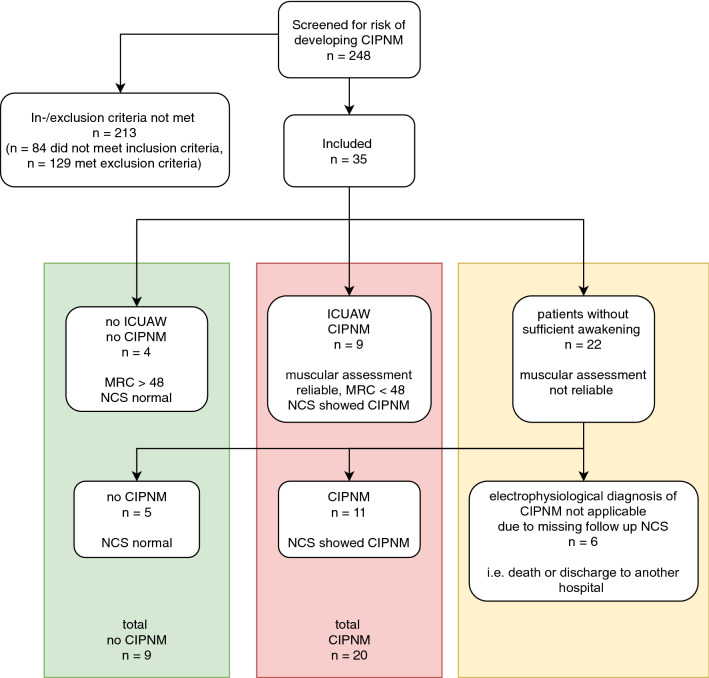
Flowchart of screened and included patients. CIPNM—Critical illness polyneuromyopathy; ICUAW—intensive care unit acquired weakness

We consecutively screened 248 intensive care unit patients. Of these, 84 did not meet any of the inclusion criteria. Further 129 patients were excluded according to above mentioned criteria. The most common causes for exclusion were preexisting diabetes mellitus (*n* = 49), excessive alcohol consumption (*n* = 35) and previous chemotherapy (*n* = 27) or combinations of these three criteria. One patient was excluded due to HbA1c > 8.5% without prior diagnosis of diabetes mellitus. We included 35 patients in the study (supplementary Table 2). Of these, four patients had a score of > 48 in MRC sum score during the whole follow-up time; therefore, diagnosis of ICUAW could be excluded by muscular assessment (Fig. 1). Nerve conduction studies were normal in these four patients. In nine patients, muscular assessment revealed clinical diagnosis of ICUAW. In these, CIPNM could be confirmed via nerve conduction studies. Of the 35 patients, 22 (65%) did not reach a sufficient level of consciousness for a reliable muscular assessment during intensive care unit treatment. Electrophysiological testing was used to diagnose CIPNM in these patients (for details see Fig. 1). Overall, CIPNM was diagnosed in 20 of the 35 patients and was excluded in nine. Electrophysiology in addition to clinical diagnosis of ICUAW helped to reduce the number of patients who were not assessable from 22 of 35 (65%) to 6 of 35 (17%).

Patient’s baseline characteristics are shown in Table 2. Demographics did not differ between CIPNM and no CIPNM patients. No significantly differences regarding disease characteristics like clinical scores at inclusion and occurrence of sepsis, resuscitation and central nervous system disease or sedation-days and vasopressor use were found (Table 2). Infections of any kind were frequent in both groups without differences between patients with and without CIPNM. Also, we found no statistically significant differences in frequency of sepsis although sepsis seemed to occur more often in CIPNM patients (Table 2). SOFA score, as well as common laboratory values for infections and inflammation like leukocyte count, C-reactive protein and procalcitonin did not differ significantly between both groups at inclusion (Table 2) or in longitudinal course (data not shown). Median follow-up time was 26 days (IQR 46). Median disease duration from admission to intensive care unit until inclusion was 6 days (IQR 8).

**Table 2:** Baseline characteristics and outcome of study population

	All patients (*n* = 29)	CIPNM (*n* = 20)	No CIPNM (*n* = 9)	*p*
*Demographics*				
Age in years, mean (SD)	65 (12)	65 (13)	65 (13)	
Women, *n* (%)	10 (35)	7 (35)	3 (33)	
BMI, mean (SD)	25 (5)	24 (5)	25 (5)	
*Disease characteristics*				
Disease duration until inclusion in days, median (IQR)	6 (8)	7 (9)	4 (10)	
Days of sedation, median (IQR)	12 (15)	14 (15)	7 (17)	
Days of vasopressor use, median (IQR)	8 (13)	10 (11)	3 (21)	
mRS at inclusion, median (IQR)	5 (0)	5 (0.2)	4.9 (0.3)	
GCS at inclusion, median (IQR)	3 (2)	3 (1)	3 (5)	
SOFA at inclusion, median (IQR)	9 (4)	11 (8)	8 (4)	
Maximum SOFA during ICU stay, median (IQR)	10 (3)	10 (2)	9 (5)	
Sepsis, *n* (%)	13 (45)	11 (55)	2 (22)	
Reanimation, *n* (%)	13 (45)	9 (45)	4 (44)	
Stroke or intracranial hemorrhage, *n* (%)	8 (28)	4 (20)	4 (44)	
Any CNS disease, *n* (%)	19 (66)	7 (35)	6 (67)	
Follow-up time in days, median (IQR)	26 (46)	38 (47)	19 (16)	
*Laboratory values at inclusion, mean (SD)*				
Leukocytes in 1000/µl	10.9 (4.7)	11.1 (5.1)	10.4 (3.8)	
Thrombocytes in 1000/µl	250 (142)	284 (156)	175 (60)	
Hemoglobin in g/dl	10.4 (2.6)	10.6 (2.6)	9.8 (2.6)	
Creatine kinase in U/l	1048 (2359)	1103 (2746)	925 (1240)	
Procalcitonin in ng/ml	1.9 (5.0)	2.4 (6.1)	1.0 (1.7)	
Glucose in mg/dl	156 (75)	166 (86)	135 (38)	
C-reactive protein in mg/l	122 (101)	106 (91)	155 (117)	
Creatinine in mg/dl	1.1 (0.8)	1.0 (0.7)	1.4 (0.9)	
Bilirubin in mg/dl	0.7 (0.8)	0.6 (0.6)	0.9 (1.0)	
paO2 in mmHg	109 (35)	111 (28)	105 (49)	
*Outcome parameters*				
Death, *n* (%)	11 (38)	7 (35)	4 (44)	n.s.
Outcome mRS, median (IQR)	5 (2)	5 (1)	5 (1)	n.s.
Outcome GCS, median (IQR)	7 (10)	7 (11)	8 (10)	n.s.
Duration of stay at ICU in days, median (IQR)	22 (21)	30 (17)	18 (8)	0.07
Duration of stay in hospital in days, median (IQR)	30 (30)	37 (20)	26 (16)	n.s.
Duration of ventilation in days, median (IQR)	11 (18)	15 (23)	9 (5)	**0.01
Duration from mechanical ventilation to assisted spontaneous ventilation in days, median (IQR)	6 (3)	7 (5)	5 (6)	*0.04
Tracheostoma, *n* (%)	12 (41)	11 (55)	1 (11)	*0.03

*BMI* Body mass index; *CIPNM* Critical illness polyneuromyopathy; *CNS* Central nervous system; *GCS* Glasgow coma scale; *ICU* Intensive care unit; *mRS* modified Rankin Scale; *pO2* Oxygen partial pressure; *SOFA* Sepsis-related organ failure assessment score

Clinical outcome regarding death, modified Rankin Scale and Glasgow coma scale was poor in both groups with a high mortality rate between 35% and 44% and a median modified Rankin Scale of 5 in both groups (Table 2). CIPNM patients had a longer duration of ventilation (*p* = 0.01**) and a longer weaning time (*p* = 0.04*) than patients without CIPNM. Also, tracheotomy was more frequently performed in CIPNM (*p* = 0.03*). Duration of intensive care unit stay was not significantly different but showed a tendency toward a longer duration of stay in CIPNM patients (*p* = 0.07, Table 2). A long-term follow-up after 6 months was only available in four patients due to poor outcome in both groups.

### Electrodiagnostics

Eleven of 20 CIPNM patients (55%) and seven of nine patients without CIPNM (78%) had amplitudes below lower limit of normal in the nerve conduction studies at inclusion. The difference between patients with and without CIPNM was not statistically significant. Many of our patients had edema of the extremities. Of the patients with reduced amplitudes at baseline examination without CIPNM, none had spontaneous activity in needle electromyography.

**Fig. 2:**
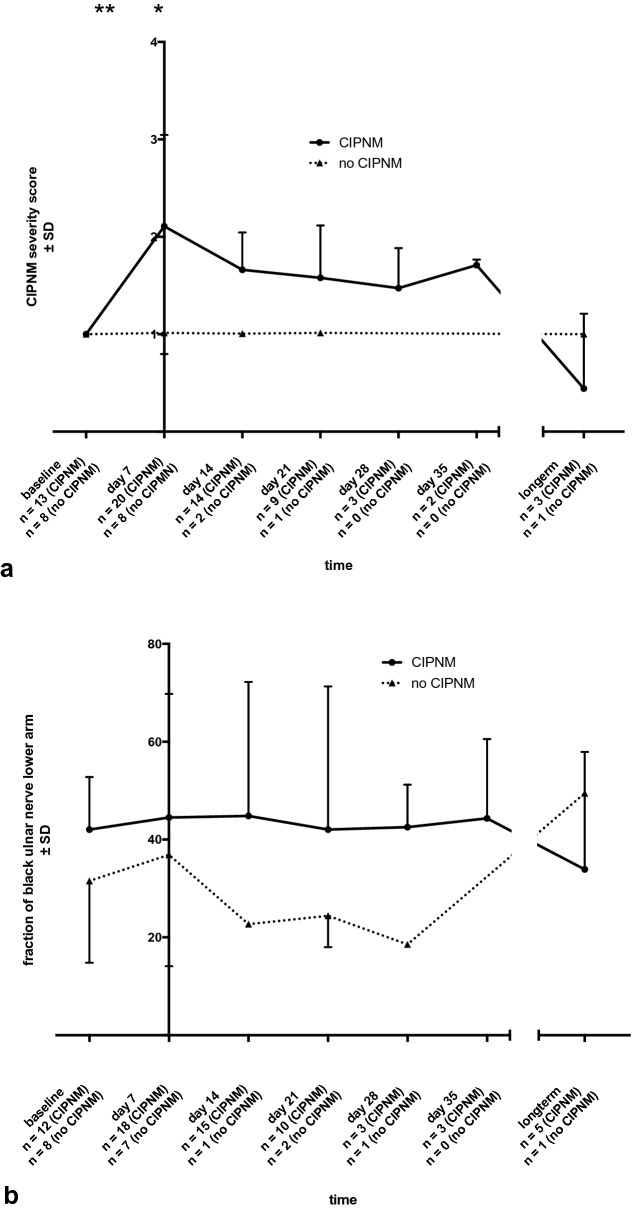
**a** CIPNM severity score derived from nerve conduction study and needle electromyography data for CIPNM and no CIPNM patients in the longitudinal course, showing the electrophysiological severity of the CIPNM with a peak 7 days after admission. **b** Fraction of black representing echogenicity of peripheral nerves for ulnar nerve at lower arm (*p* = 0.03* for Mann–Whitney *U*-test for the whole intensive care unit stay), showing hypoechoic nerves in CIPNM as a possible marker for edema and inflammation

For visualization of CIPNM severity in disease course, CIPNM severity score is shown in Fig. 2a. This score showed a peak of CIPNM 7 days after inclusion.

Needle electromyography could overall be performed in 22 patients on different time points. Of these patients, seventeen had CIPNM and five had no CIPNM. Of the CIPNM patients, 15 out of 17 (88%) had spontaneous activity in needle electromyography during intensive care unit stay, while none of the patients without CIPNM had spontaneous activity in needle electromyography. As needle electromyography was not feasible at every visit due to anticoagulation in many patients, reliable analysis of needle electromyography changes in disease course was not possible.

### High-Resolution Ultrasound

At baseline, we found no statistically significant differences of cross-sectional area between both groups.

**Fig. 3:**
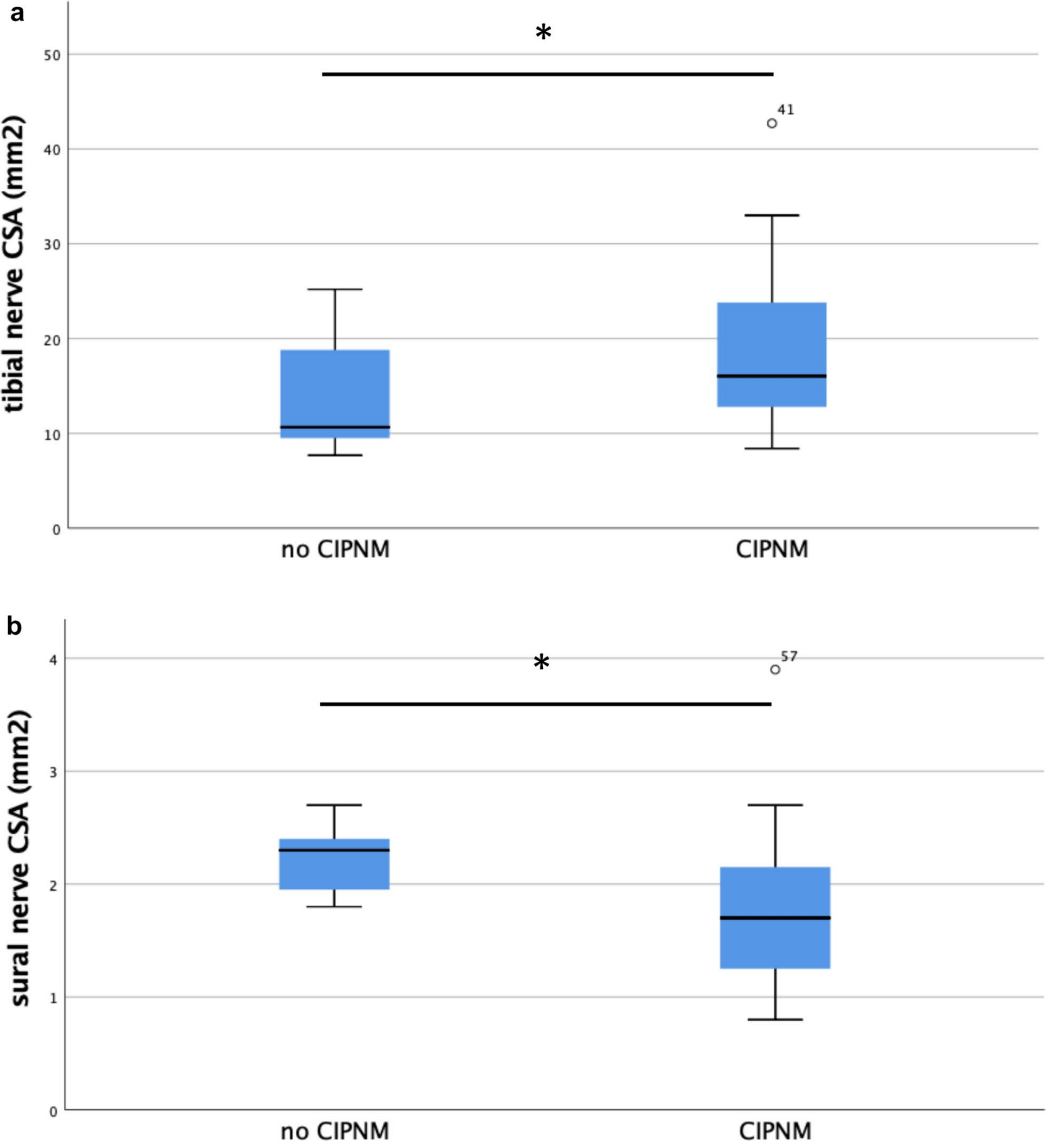
Distribution of cross-sectional area of tibial (**a**) and sural (**b**) nerve at day 7 for both groups. In CIPNM patients, the tibial nerve in the popliteal fossa was larger (*p* = 0.04) and the sural smaller (*p* = 0.01) than in patients without CIPNM

On follow-up day seven, at the peak of CIPNM, the mean cross-sectional area of tibial nerve in the popliteal fossa of CIPNM patients was higher than that of patients without CIPNM (*p* = 0.04*). Moreover, CIPNM patients had a smaller cross-sectional area of the sural nerve compared to patients without CIPNM (*p* = 0.01**, Table 3, Fig. 3). These differences in tibial and sural nerve were statistically significant, but cross-sectional area of sural nerve in both groups was still within the range of reference values of healthy persons.

**Table 3:** High-resolution ultrasound data at baseline and at CIPNM peak (day 7)

	All patients (*n* = 29)	CIPNM (*n* = 20)	No CIPNM (*n* = 9)	*p*
*At baseline*				
Median nerve CSA in mm^2^, mean (SD)				
Carpal tunnel	11.5 (3.1)	11.7 (3.3)	11.3 (3.0)	
Lower arm	7.8 (2.2)	7.5 (2.0)	8.2 (2.5)	
Upper arm	10.3 (3.4)	9.8 (3.4)	11.1 (2.6)	
Ulnar nerve CSA in mm^2^, mean (SD)				
Guyon Loge	5.5 (1.5)	5.4 (1.5)	5.8 (1.5)	
Lower arm	5.8 (1.5)	5.8 (1.7)	5.8 (1.3)	
Elbow	8.6 (2.7)^a^	8.0 (2.5)	9.5 (2.9)^a^	
Upper arm	6.1 (2.1)	6.1 (2.2)	6.2 (2.1)	
Radial nerve CSA in mm^2^, mean (SD)				
Spiral groove	4.6 (1.6)	4.8 (1.8)	4.1 (1.1)	
Fibular nerve CSA in mm^2^, mean (SD)				
Fossa poplitea	6.8 (2.2)	6.7 (1.9)	7.1 (2.7)	
Fibular head	12.5 (4.0)^a^	11.7 (4.1)	13.9 (3.7)^a^	
Tibial nerve CSA in mm^2^, mean (SD)				
Fossa poplitea	16.7 (7.3)^a^	17.4 (7.9)^a^	15.4 (6.1)^a^	
Malleolar	8.5 (3.8)	8.6 (3.9)	8.2 (3.5)	
Sural nerve CSA in mm^2^, mean (SD)				
Lower leg	2.0 (0.8)	2.0 (0.9)	2.0 (0.5)	
Vagal nerve CSA in mm^2^, mean (SD)				
Vagina carotica	2.1 (0.7)	2.0 (0.7)	2.1 (0.7)	
Tibial anterior muscle				
Heckmatt Score, median (IQR)	2 (1)	1.5 (1)	2 (1)	
*At CIPNM peak (day 7)*				
Median nerve CSA in mm^2^, mean (SD)				
Carpal tunnel	11.4 (3.1)	11.0 (3.0)	12.3 (3.4)	n.s.
Lower arm	8.0 (2.8)	8.1 (2.7)	7.9 (3.1)	n.s.
Upper arm	9.3 (2.9)	9.0 (2.5)	10.1 (3.6)	n.s.
Ulnar nerve CSA in mm^2^, mean (SD)				
Guyon Loge	5.2 (1.8)	5.1 (1.9)	5.3 (1.4)	n.s.
Lower arm	5.8 (1.4)	5.6 (1.3)	6.4 (1.6)	n.s.
Elbow	8.6 (2.5)^a^	8.3 (2.6)^a^	9.5 (2.2)^a^	n.s.
Upper arm	6.5 (2.0)	6.3 (2.1)	7.1 (2.3)	n.s.
Radial nerve CSA in mm^2^, mean (SD)				
Spiral groove	4.6 (1.7)	4.6 (1.6)	4.8 (2.0)	n.s.
Fibular nerve CSA in mm^2^, mean (SD)				
Fossa poplitea	6.9 (2.4)	6.7 (1.7)	7.3 (3.9)	n.s.
Fibular head	12.5 (4.9)^a^	12.3 (5.0)^a^	13.0 (4.9)^a^	n.s.
Tibial nerve CSA in mm^2^, mean (SD)				
Fossa poplitea	17.3 (7.8)^a^	18.5 (7.9)^a^	13.7 (6.6)	*0.04
Malleolar	8.0 (4.0)	7.6 (3.9)	9.2 (4.2)	n.s.
Sural nerve CSA in mm^2^, mean (SD)				
Lower leg	1.9 (0.6)	1.7 (0.6)	2.2 (0.3)	**0.01
Vagal nerve CSA in mm^2^, mean (SD)				
Vagina carotica	2.1 (1.1)	2.1 (1.1)	2.1 (1.0)	n.s.
Tibial anterior muscle				
Heckmatt Score, median (IQR)	1.5 (0.5)	1.5 (0.5)	2 (0.5)	n.s.

Values marked with an ^a^ are above the normal values of our ultrasound laboratory

*CIPNM* critical illness polyneuromyopathy; *CSA* cross-sectional area

**Fig. 4:**
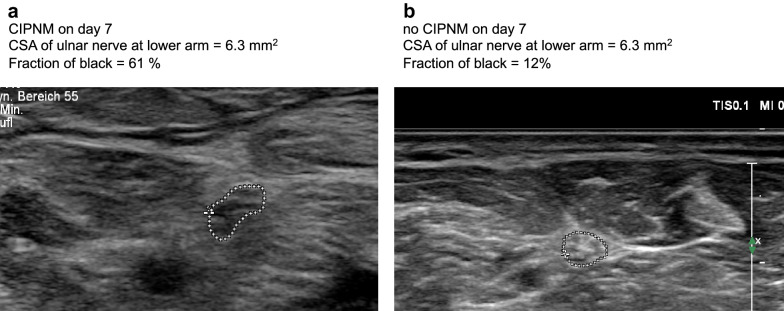
Sample images of ulnar nerve of one CIPNM patient (**a**) and one patient without CIPNM (**b**) at CIPNM peak, showing hypoechogenic nerves in CIPNM compared to patient without CIPNM

Fraction of black was higher in patients with CIPNM compared to patients without CIPNM during the entire intensive care unit stay (*p* = 0.03* for median nerve at forearm, *p* = 0.03* for ulnar nerve at forearm, *p* = 0.04* for fibular nerve, exemplarily shown for ulnar nerve at forearm in Figs. 2b and 4, Mann–Whitney *U*-test for the whole intensive care unit stay). CIPNM patients had a higher fraction of black already at the first examination at admission to intensive care unit than patients without CIPNM. As example in Figs. 2b and 4, we show the data for the ulnar nerve.

Heckmatt score of tibial anterior muscle was not significantly different between both groups.

All ultrasound data were collected and analyzed as exploratory data.

### Cytokine Results

At baseline, no significant differences in cytokine levels between both groups were found.

At peak of CIPNM (day 7), a statistically significant increase of GM-CSF, IL20 and MIP1 alpha was detectable (*p* = 0.04* each, t test), possibly indicating monocyte and macrophage activation at the peak of CIPNM.

**Fig. 5:**
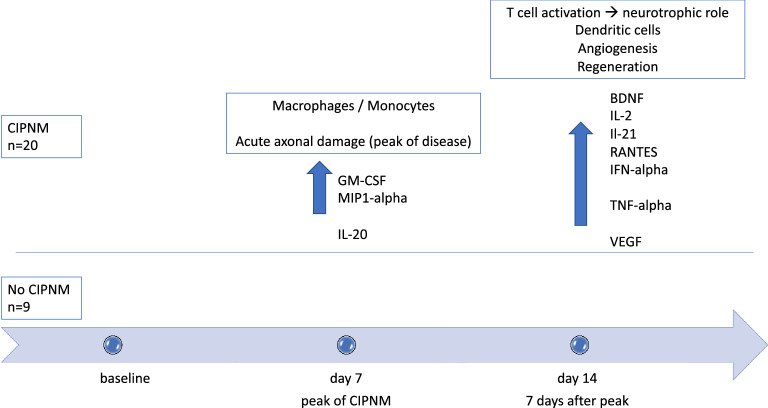
Conclusions from the cytokine analyses regarding possible aspects of pathogenesis in CIPNM

Seven days after CIPNM peak (day 14), IL2 (*p* = 0.03*), IL21 (*p* = 0.04*), CCL5/RANTES (*p* = 0.01**), IFN alpha (0.02*), TNF alpha (*p* = 0.03*) and BDNF (*p* = 0.01**) and VEGFd (*p* = 0.03*) were increased in CIPNM patients (supplementary Fig. 1). These elevated T-cell cytokines (IL2, IL21, CCL5/RANTES) and markers for macrophages (TNF alpha) and dendritic cells (IFN alpha) possibly indicate a systemic immune response, present in intensive care unit patients who developed a CIPNM. A summary of these results is shown in Fig. 5.

All cytokine data were collected and analyzed as exploratory data.

## Discussion

In this study, we found that novel approaches like neuromuscular ultrasound and cytokine analyses reveal signs of inflammation in the longitudinal course of electrophysiologically primarily axonal CIPNM.

At the peak of CIPNM, we found signs of monocyte and macrophage activation in cytokine analyses, which have so far rarely been described [15], but are known in autoimmune inflammatory neuropathies [24]. Simultaneously enlargement of the tibial nerve in ultrasound as well as hypoechogenicity of nerves can be considered as signs of an acute inflammation in ultrasound. Hypoechoic nerves in other inflammatory neuropathies, i.e., chronic inflammatory demyelinating polyneuropathy are supposed to correlate to inflammation, in particular infiltration of inflammatory cells, edema and onion bulbs, whereas chronic irreversible axonal damage with scar tissue or fibrosis is regarded to result in hyperechoic alterations [21, 25]. Therefore, our finding of hypoechoic nerves in CIPNM suggests that edema and inflammation of peripheral nerves may occur within the first weeks of CIPNM.

On follow-up day 14, seven days after CIPNM peak, the cytokine measurements showed signs of a systemic immune response and cytokine profiles associated to neovascularization (VEGFd) and regeneration (BDNF). Regeneration was also observed in the CIPNM severity score in those patients who were available for long-term follow-up a few months after intensive care unit treatment.

SOFA score, as well as common laboratory values for infections and inflammation, did not differ significantly between both groups, so systemic infection should not be the cause for differences in cytokine analyses.

Diagnosis and monitoring of ICUAW is challenging, especially in the early days of an intensive care unit, stay as assessment of neuromuscular symptoms is impaired in analgosedated patients and diagnosis relies on the exclusion of other diseases and electrophysiological measurements. Electrophysiology in our study was helpful to diagnose CIPNM as many patients did not reach a sufficient level of consciousness for muscular assessment in our study. Our data show that in patients, in which actually polyneuropathy-causing preexisting diseases were excluded through inclusion and exclusion criteria, a single nerve conduction study detects reduced amplitudes in several patients. We reckon that this does not always reflect a real polyneuropathy or CIPNM. Prevalence of polyneuropathies in the general population between 60 and 70 years of age was 8% previously reported in a Dutch study with approximately 50% of these cases newly diagnosed [26]. However, this prevalence was observed in the general population, not in ICU patients. At ICU, amplitudes of NCS are influenced through conditions like edema which lead to falsely reduced amplitudes in all patients. Therefore, it is most likely that patients did not actually have an unknown preexisting polyneuropathy at baseline but issues like edema caused low amplitudes. Other studies reported reduced amplitudes in nerve conduction studies in ICU patients compared to healthy controls independently from CIPNM [6]. The reference values for amplitudes for ICU patients were reported lower than that for healthy controls probably due to reduction in muscle or nerve excitability or edema. This is also supported by the fact that reduced amplitudes in the first examination were found equally in CIPNM and in non-CIPNM patients and also by the fact that none of the patients without CIPNM but with reduced amplitudes at baseline had spontaneous activity in needle electromyography as a sign of axonal damage. Moreover, we did not exclude patients with reduced amplitudes at baseline as it is still not known how quickly CIPNM develops in ICU patients. The suspected metabolic, ischemic and inflammatory mechanisms can lead to a reduced function of the peripheral nerves within a few hours and days, i.e., in severe sepsis [13]. As a median of 6 days from admission to inclusion in the study passed, we did not want to miss an already developed early CIPNM at the timepoint of the first examination. Therefore, these patients were followed up in their whole course to confirm or rule out further progression over time. Accordingly, certain diagnosis of CIPNM is only possible if the evolution of a polyneuropathy during intensive care unit stay is proven. Therefore, in our definition of CIPNM we used evolution of nerve conduction studies and needle electromyography parameters during intensive care unit stay.

Supporting the reliability of the diagnostic criteria for CIPNM used in this study, the outcome of the CIPNM group regarding ventilation duration was significantly worse than that of the non-CIPNM patients, which is in line with the literature [2, 3, 27]. Also the incidence of CIPNM (69%) in our study group is similar to those reported in literature [2, 3, 27]. The clinical outcome measured by modified Rankin Scale and the portion of deceased patients was not different between CIPNM and non-CIPNM group. Probably this is due to the fact that all patients were severely ill and had a poor outcome, not just those who additionally developed a CIPNM. For future studies, it would be interesting to also examine less severely ill patients. Also, longitudinal needle electromyography studies would be desirable, but could not be performed in this study due to contraindications.

International electrophysiological consensus criteria for diagnosis of CIPNM do not exist. Axonal degeneration in CIPNM can be demonstrated in fibular nerves more than in others^17^; therefore, we used nerve conduction studies of fibular nerve for definition and longitudinal assessment. We developed an electrophysiological score that allows detection of worsening in the nerve conduction studies longitudinally.

A major strength of this study is that our study is the first thorough longitudinal study simultaneously investigating pathophysiological characteristics of CIPNM and diagnostic features through the ‘gold standard’ of nerve conduction studies longitudinally. Knowledge about pathophysiology of CIPNM is a prerequisite for the development of new treatment options. Our findings provide first evidence for further research on anti-inflammatory/immunomodulatory treatment for CIPNM. Until now, immunomodulatory therapies were not found to be effective in CIPNM, although studies specifically looking at the CIPNM are rare [28]. In sepsis patients, steroid therapy has been recommended in patients with adrenergic insufficiency, but less due to the immunomodulatory effects [29–31]. The TNF alpha activation in our CIPNM patients provides the thesis of a potential beneficial effect of corticosteroids in these patients [32, 33]. Also, anti-inflammatory therapies in combination with neuroprotection and reduction of oxidative stress could be promising.

As clinical impact, our results show that CIPNM can be safely diagnosed only by repeated nerve conduction studies during the first week on intensive care unit stay. This is crucial as the early ‘inflammatory’ phase of CIPNM pathophysiology implies that an early diagnosis and treatment will be essential to reduce CIPNM incidence and severity in the future.

However, this study has some important limitations: An important limitation of our study is the small sample size. Particularly, our findings in long-term course are limited due to small sample size and must be interpreted with caution. Overall, ultrasound and cytokine data were conducted and analyzed as exploratory data; therefore, the results need confirmation in further studies. As no other authors described, the ultrasound alterations in CIPNM with a similar study protocol and similar cytokine analyses, we could not perform sample size calculation. Alterations of cross-sectional area were overall small and could possibly also result from technical or examiner-dependent influences. Especially the cross-sectional area of the sural nerve at the peak of CIPNM was reduced statistically significantly in CIPNM patients, but the cross-sectional area of both groups was within normal values and differences were only small. A reduced cross-sectional area in other neuropathies is regarded resulting from axonal damage, but it is unlikely that nerve atrophy develops within 7 days in CIPNM. Instead, in CIPNM inflammation could occur with different celerity in different nerve sections, i.e., sensory nerves like the sural nerve could be affected by inflammation in the early hours/days and other nerves like the tibial nerve later, which is why we would find different results in both nerves and a cross-sectional area in the sural nerve that already normalized again or even reduced. However, as cross-sectional area alterations were small, we cannot substantiate these considerations with our data sufficiently, so that further studies are necessary to assess this more precisely. Currently, nerve ultrasound cannot be recommended as diagnostic tool in clinical practice, confirming Witteveen et al. [10]. We included patients with central nervous system diseases and spastic hemiparesis or inactivity atrophy could influence nerve conduction studies and muscle ultrasound. Also, central paresis due to central nervous system disease influence muscle strength assessment. Furthermore, as median time from intensive care unit admission to baseline examination was 6 days, we were unable to examine the early phase of the CIPNM. For future studies, cytokine profiling would be interesting in this earlier phase in order to find biomarkers, which would distinguish between CIPNM and non-CIPNM patients. Overall, our study is rather a basis for future research and should not be used to conclude specific generalized diagnostic recommendations.

## Conclusion

Exploratory neuromuscular ultrasound and cytokine analyses showed signs of inflammation like macrophage and monocyte activation at the peak of CIPNM followed by a systemic immune response occurring parallel to axonal damage. This underlines the role of both axonal damage and inflammation in pathogenesis of CIPNM. As clinical impact, our study shows that repeated nerve conduction studies should be performed to make a reliable diagnosis of CIPNM.

## Electronic supplementary material

Below is the link to the electronic supplementary material.

Supplementary material 1 (DOCX 129 kb)

Supplementary Figure 1: Longitudinal cytokine levels for CIPNM and no CIPNM patients, showing an activation of GM-CSF, IL20 and MIP1 alpha at the peak of CIPNM; an activation of IL2, IL21, CCL5/RANTES, IFN alpha and TNF alpha as well as BDNF and VEGFd 7 days after CIPNM peak. (PDF 47 kb)

Supplementary Figure 2: Scatter diagram of CIPNM severity score and cytokines which showed positive correlation in correlation analysis (corresponding to supplementary Table 3). A: IL2, B: IL21, C: CCL5/RANTES, D: BDNF, E: VEGFd, F: IFN alpha, G: TNF alpha. (PDF 670 kb)

## Data Availability

The datasets used and/or analyzed during the current study are available from the corresponding author on reasonable request.

## Abbreviations

ICUAW: Intensive care unit acquired weakness
CIPNM: Critical illness polyneuromyopathy
BDNF: Brain-derived neurotrophic factor
CCL: C-C motif chemokine ligand
PECAM: Platelet endothelial cell adhesion molecule
CXCL: C-X-C motif chemokine ligand
GM-CSF: Granulocyte-macrophage colony-stimulating factor
HGF: Hepatocyte growth factor
IFN: Interferon
IL: Interleukin
LIF: Leukemia inhibitory factor
OPG: Osteoprotegerin
SCF: Stem cell factor
TNF: Tumor necrosis factor
t-PA: Tissue plasminogen activator
TSLP: Thymic stromal lymphopoietin
VEGF: Vascular endothelial growth factor
PDGF: Platelet-derived growth factor
