# Supplementary material for: New Approaches to Critical Illness Polyneuromyopathy: High-Resolution Neuromuscular Ultrasound Characteristics and Cytokine Profiling
Source: Neurocrit Care. 2020 Nov 24;35(1):139–52. doi: 10.1007/s12028-020-01148-2 (PMC7685687; doi:10.1007/s12028-020-01148-2)
Supplement: Supplementary file 1 — Supplementary material 1 (DOCX 129 kb) [file 12028_2020_1148_MOESM1_ESM.docx]

| **Supplementary table 1. Cytokines included in multiplex assay panel** | |
| --- | --- |
| - Interleukin 12 (IL-12p70)  - Brain-Derived Neurotrophic Factor (BDNF)  - C-C motif chemokine ligand 11 (CCL11, eotaxin-1)  - C-C motif chemokine ligand 3 (CCL3, MIP-1 alpha)  - C-C motif chemokine ligand 3 (CCL4, MIP-1 beta)  - C-C motif chemokine ligand 5 (CCL5, RANTES)  - Platelet endothelial cell adhesion molecule (PECAM-1, CD31)  - P-Selectin (CD62P)  - C-X-C motif chemokine ligand 1 (CXCL1, GRO alpha)  - C-X-C motif chemokine ligand 10 (CXCL10, IP-10)  - Granulocyte-macrophage colony-stimulating factor (GM-CSF)  - Hepatocyte growth factor (HGF)  - Interferon alpha (IFN alpha)  - Interferon gamma (IFN gamma)  - Interleukin-1 receptor antagonist (IL-1RA)  - Interleukins: IL-1 alpha, IL-1 beta, IL-10, IL-13, IL-15, IL-16, IL-17A, IL-2, IL-20, IL-21,  IL-4, IL-5, IL-6, IL-7, IL-8 (CXCL8), IL-9  - Leukemia inhibitory factor (LIF)  - Osteoprotegerin (OPG)  - Soluble TNF receptor type II (sCD120b, sTNF-RII)  - Stem Cell Factor (SCF)  - Tumor necrosis factor alpha (TNF alpha)  - Tissue plasminogen activator (t-PA)  - Thymic stromal lymphopoietin (TSLP)  - Vascular endothelial growth factor A (VEGF-A)  - Vascular endothelial growth factor D (VEGF-D)  - Platelet-derived growth factor subunit B (PDGF-BB)  - Monocyte chemoattractant protein 2 (MCP-2, CCL8) |  |

| **Supplementary table 2. Detailed characteristics of study population** | | | | | | |  |  |  |  |  |
| --- | --- | --- | --- | --- | --- | --- | --- | --- | --- | --- | --- |
| ID | Group  (1 - CIPNM,  0 - no CIPNM) | Gender | Age (years) | Height (cm) | Weight (kg) | Diagnosis | Death | Cardiopulmonary resuscitation | Sepsis | Disease duration until inclusion (days) * | Follow up time (days) |
| 1 | 1 | female | 79 | 165 | 55 | NSTEMI, asystole | 0 | 1 | 0 | 2 | 153 |
| 2 | 1 | female | 62 | 170 | 60 | Urosepsis | 0 | 0 | 1 | 7 | 18 |
| 3 | 0 | male | 45 | 175 | 75 | STEMI, cardiogenic shock | 0 | 0 | 0 | 4 | 19 |
| 4 | 1 | female | 33 | 170 | 55 | Meningoencephalitis | 0 | 0 | 0 | 5 | 117 |
| 5 | 1 | male | 64 | 175 | 80 | Pneumococcal sepsis | 0 | 0 | 1 | 57 | 163 |
| 6 | 1 | male | 62 | 180 | 80 | Ventricular fibrillation | 0 | 1 | 0 | 1 | 151 |
| 7 | n.a. |  |  |  |  |  |  |  |  |  |  |
| 8 | 1 | female | 64 | 160 | 45 | Multiple cerebral infarctions, suspected angiitis of CNS | 1 | 0 | 1 | 37 | 68 |
| 9 | 1 | male | 60 | 170 | 110 | Cerebral infarction | 0 | 0 | 1 | 7 | 42 |
| 10 | 1 | male | 67 | 180 | 80 | Cardiopulmonary resuscitation, cerebral edema | 1 | 1 | 0 | 2 | 8 |
| 11 | 0 | male | 70 | 170 | 70 | Acute kidney injury, acute exacerbation of COPD | 0 | 0 | 1 | 4 | 145 |
| 12 | n.a. |  |  |  |  |  |  |  |  |  |  |
| 13 | n.a. |  |  |  |  |  |  |  |  |  |  |
| 14 | 1 | male | 69 | 170 | 50 | Pneumococcal sepsis | 1 | 0 | 1 | 7 | 41 |
| 15 | 0 | male | 83 | 180 | 80 | Cardiopulmonary resuscitation, Staphylococcus aureus sepsis | 1 | 1 | 1 | 18 | 26 |
| 16 | n.a. |  |  |  |  |  |  |  |  |  |  |
| 17 | 1 | male | 82 | 180 | 80 | Epileptic seizure with cardiopulmonary resuscitation | 1 | 1 | 0 | 1 | 9 |
| 18 | 0 | female | 48 | 170 | 65 | Epileptic seizure with aspiration pneumonia | 0 | 0 | 0 | 6 | 10 |
| 19 | 0 | male | 73 | 175 | 75 | Multiple cerebral infarctions, cardiopulmonary resuscitation | 0 | 1 | 0 | 3 | 25 |
| 20 | 1 | male | 76 | 180 | 80 | Pneumococcal sepsis, acute hypercapnic respiratory failure | 0 | 0 | 1 | 7 | 34 |
| 21 | 0 | female | 66 | 165 | 60 | Multiple cerebral infarctions, suspected angiitis of CNS | 1 | 0 | 0 | 0 | 5 |
| 22 | 1 | male | 53 | 180 | 80 | STEMI, cardiogenic shock, cardiopulmonary resuscitation | 0 | 1 | 0 | 1 | 7 |
| 23 | 0 | male | 61 | 180 | 80 | NSTEMI, cardiogenic shock | 1 | 1 | 0 | 4 | 10 |
| 24 | 0 | female | 79 | 165 | 65 | Cardiopulmonary resuscitation | 1 | 1 | 0 | 18 | 26 |
| 25 | 1 | male | 55 | 180 | 75 | STEMI, cardiogenic shock, cardiopulmonary resuscitation | 0 | 1 | 1 | 6 | 60 |
| 26 | 1 | female | 51 | 165 | 40 | Pneumonia, choreatiform movement disorder with dysphagia | 0 | 0 | 0 | 7 | 22 |
| 27 | 1 | male | 82 | 180 | 95 | Ventricular fibrillation | 1 | 1 | 0 | 7 | 19 |
| 28 | 0 | male | 57 | 180 | 120 | Cerebral infarction | 0 | 0 | 0 | 1 | 12 |
| 29 | n.a. |  |  |  |  |  |  |  |  |  |  |
| 30 | 1 | female | 58 | 165 | 90 | Cardiopulmonary resuscitation | 0 | 1 | 1 | 2 | 43 |
| 31 | 1 | male | 74 | 175 | 80 | Pneumonia, acute kidney injury | 0 | 0 | 1 | 18 | 26 |
| 32 | 1 | female | 63 | 160 | 65 | Seizures with lactic acidosis, hyperprolinemia | 0 | 1 | 1 | 12 | 54 |
| 33 | 1 | male | 81 | 175 | 75 | Pneumonia, acute respiratory distress syndrome | 1 | 0 | 1 | 18 | 27 |
| 34 | n.a. |  |  |  |  |  |  |  |  |  |  |
| 35 | 1 | male | 61 | 180 | 80 | Acute exacerbation of COPD, pneumonia | 1 | 0 | 0 | 0 | 6 |
| * A disease duration until inclusion with ≥ 7 days resulted from patients who did not meet our inclusion criteria at admission to ICU but had severe complications during the ICU stay (e.g. sepsis, basilar embolism), then meeting our inclusion criteria and then being in risk of developing CIPNM. For the analysis of the peak of CIPNM, the baseline day was set for each patient individually to the timepoint when we assumed the beginning risk of developing a CIPNM. Two patients (ID 5 and 8) were included after the acute phase, also this was considered in calculating peak of disease. | | | | | | | | | | | |

| **Supplementary table 3. Peak CIPNM severity score correlates with cytokine levels** | | | | |
| --- | --- | --- | --- | --- |
| **7 days after peak while maximum SOFA score shows no correlation** | | | | |
|  | **peak CIPNM severity score** |  | **maximum SOFA score** |  |
|  | Spearman correlation coefficient r | p | Spearman correlation coefficient r | p |
| IL2 ^ | 0.76 * | 0.02 | 0.10 | 0.82 |
| IL21 ^ | 0.82 ** | 0.01 | -0.02 | 0.96 |
| RANTES ^ | 0.89 ** | <0.01 | 0.12 | 0.76 |
| BDNF ^ | 0.84 ** | <0.01 | -0.16 | 0.71 |
| VEGFd ^ | 0.72 * | 0.03 | 0.04 | 0.93 |
| GMCSF ' | 0.10 | 0.70 | 0.52 | 0.04 * |
| IFNalpha ^ | 0.70 * | 0.04 | -0.33 | 0.43 |
| IL20 ' | 0.13 | 0.62 | 0.11 | 0.68 |
| MIP1alpha ' | 0.38 | 0.14 | 0.28 | 0.30 |
| TNFalpha ^ | 0.81 ** | <0.01 | -0.11 | 0.80 |
|  |  |  |  |  |
| ^ 7 days after peak |  |  |  |  |
| ' at peak |  |  |  |  |
| * level of significance |  |  |  |  |
