# Supplementary figures and images for: New Approaches to Critical Illness Polyneuromyopathy: High-Resolution Neuromuscular Ultrasound Characteristics and Cytokine Profiling
Source: Neurocrit Care. 2020 Nov 24;35(1):139–52. doi: 10.1007/s12028-020-01148-2 (PMC7685687; doi:10.1007/s12028-020-01148-2)

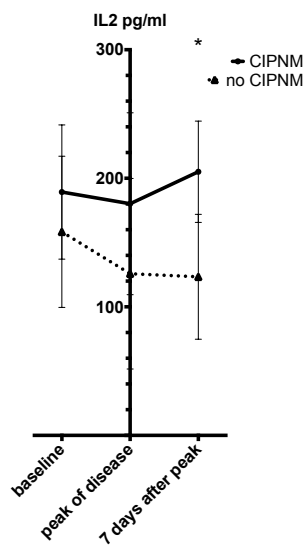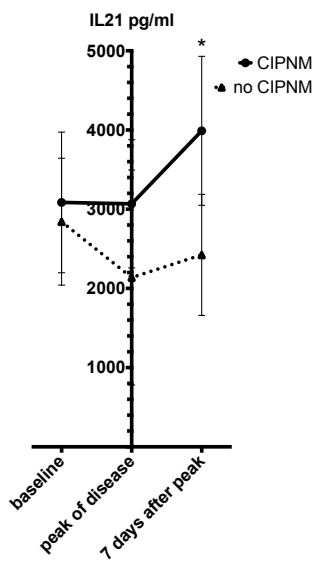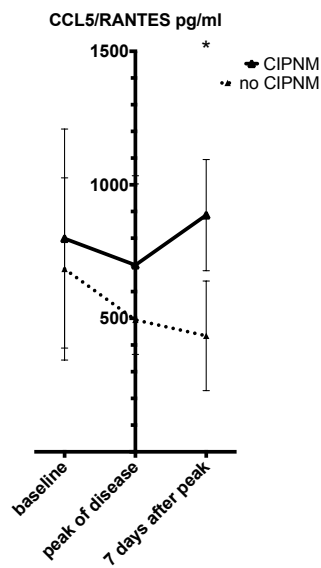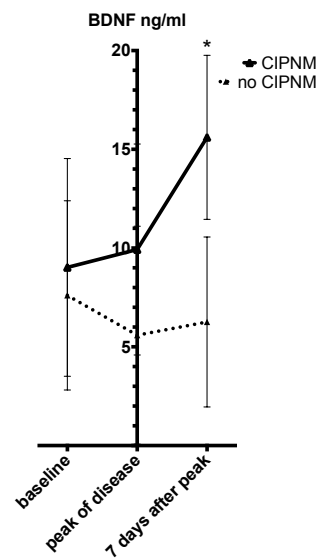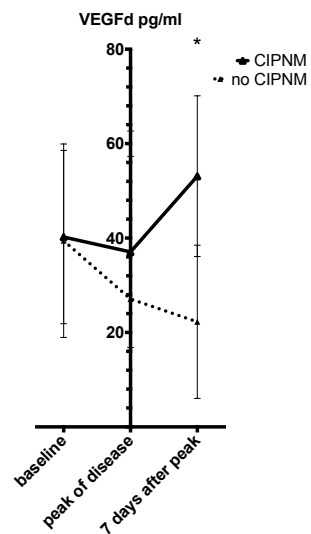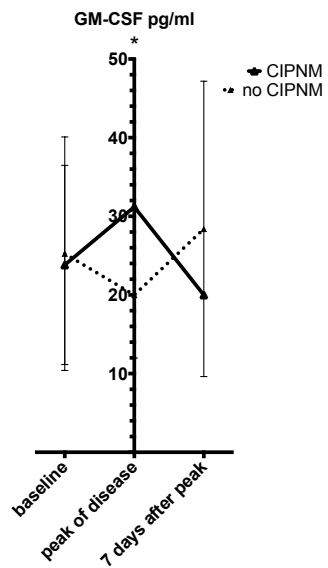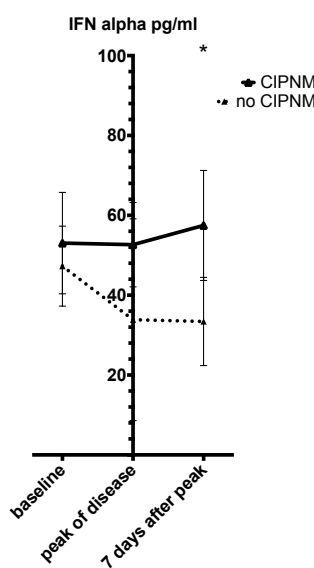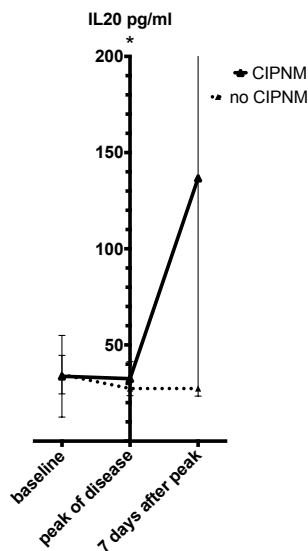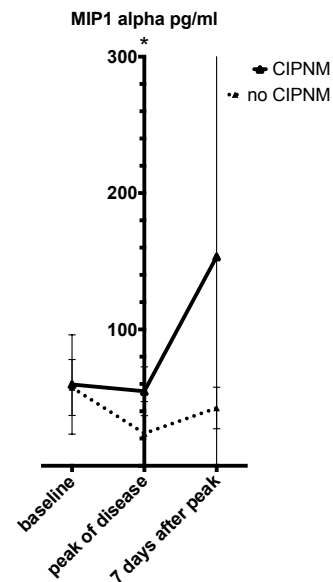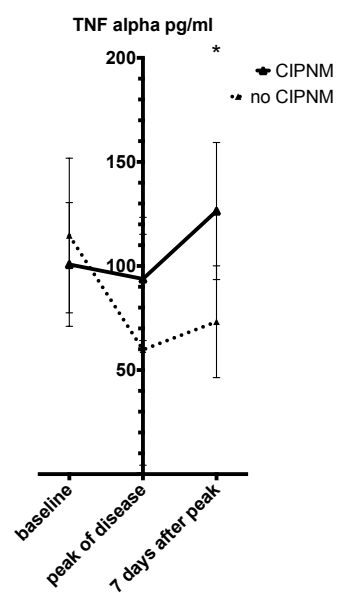

Supplement: Supplementary file 2 — Supplementary Figure 1: Longitudinal cytokine levels for CIPNM and no CIPNM patients, showing an activation of GM-CSF, IL20 and MIP1 alpha at the peak of CIPNM; an activation of IL2, IL21, CCL5/RANTES, IFN alpha and TNF alpha as well as BDNF and VEGFd 7 days after CIPNM peak. (PDF 47 kb) [file 12028_2020_1148_MOESM2_ESM.pdf]

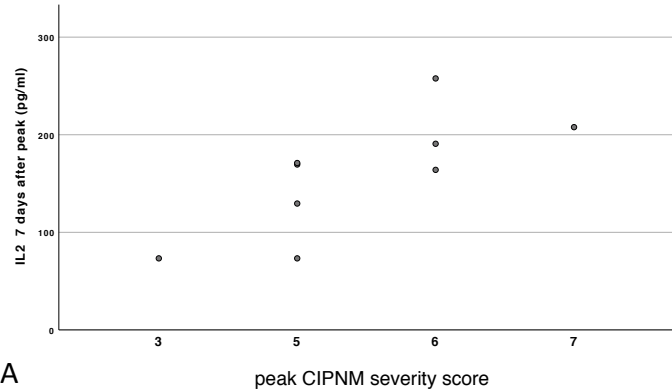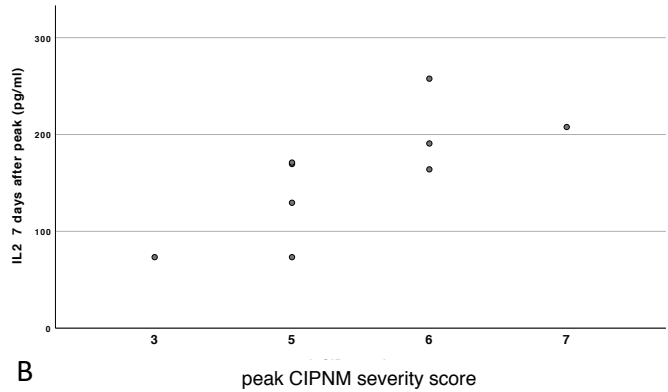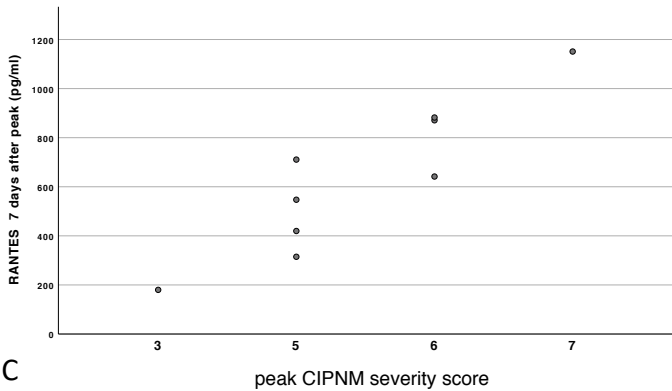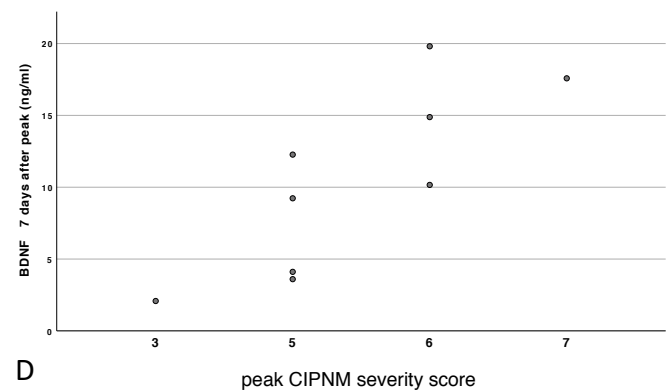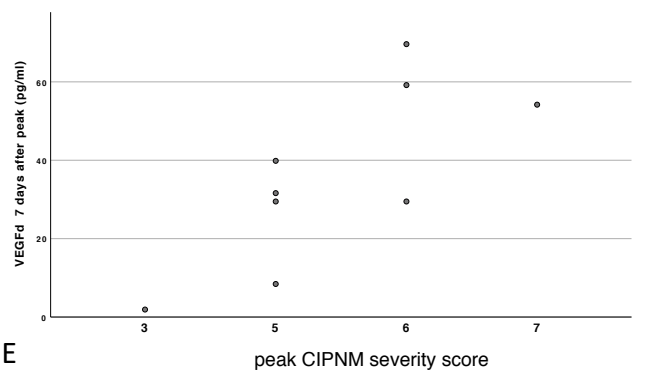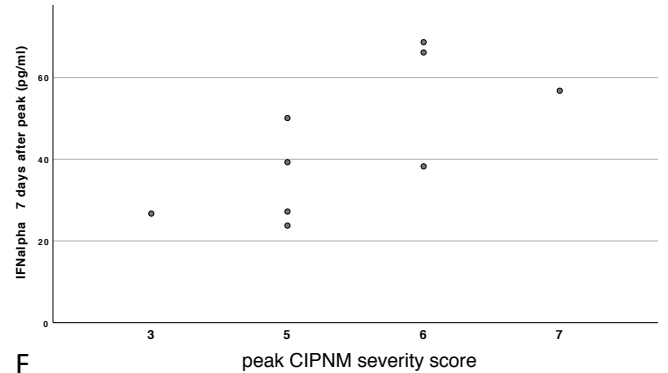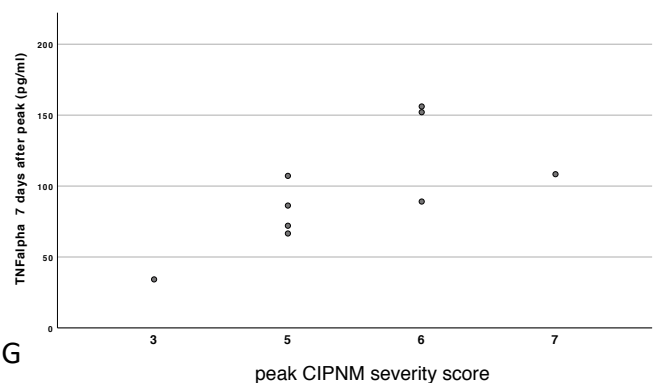

Supplement: Supplementary file 3 — Supplementary Figure 2: Scatter diagram of CIPNM severity score and cytokines which showed positive correlation in correlation analysis (corresponding to supplementary Table 3). A: IL2, B: IL21, C: CCL5/RANTES, D: BDNF, E: VEGFd, F: IFN alpha, G: TNF alpha. (PDF 670 kb) [file 12028_2020_1148_MOESM3_ESM.pdf]
